# Correlates of protection and viral load trajectories in omicron breakthrough infections in triple vaccinated healthcare workers

**DOI:** 10.1038/s41467-023-36984-1

**Published:** 2023-03-22

**Authors:** Ulrika Marking, Sebastian Havervall, Nina Greilert Norin, Oscar Bladh, Wanda Christ, Max Gordon, Henry Ng, Kim Blom, Mia Phillipson, Sara Mangsbo, Jessica J. Alm, Anna Smed-Sörensen, Peter Nilsson, Sophia Hober, Mikael Åberg, Jonas Klingström, Charlotte Thålin

**Affiliations:** 1 Department of Clinical Sciences, Karolinska Institutet Danderyd Hospital, Stockholm, Sweden; 2 Center for Infectious Medicine, Department of Medicine Huddinge, Karolinska Institutet, Stockholm, Sweden; 3 Department of Medical Cell Biology and SciLifeLab, Uppsala University, Uppsala, Sweden; 4 Department of Pharmacy and SciLifeLab, Uppsala University, Uppsala, Sweden; 5 Department of Microbiology, Tumor and Cell Biology & National Pandemic Center, Karolinska Institutet, Solna, Sweden; 6 Division of Immunology and Allergy, Department of Medicine Solna, Karolinska Institutet, Karolinska University Hospital, Stockholm, Sweden; 7 Department of Protein Science, KTH Royal Institute of Technology, SciLifeLab, Stockholm, Sweden; 8 Department of Medical Sciences, Clinical Chemistry and SciLifeLab, Uppsala University, Uppsala, Sweden; 9 Division of Molecular Medicine and Virology, Department of Biomedical and Clinical Sciences, Linköping University, Linköping, Sweden

**Keywords:** Viral infection, RNA vaccines

## Abstract

Vaccination offers protection against severe COVID-19 caused by SARS-CoV-2 omicron but is less effective against infection. Characteristics such as serum antibody titer correlation to protection, viral abundance and clearance of omicron infection in vaccinated individuals are scarce. We present a 4-week twice-weekly SARS-CoV-2 qPCR screening in 368 triple vaccinated healthcare workers. Spike-specific IgG levels, neutralization titers and mucosal spike-specific IgA-levels were determined at study start and qPCR-positive participants were sampled repeatedly for two weeks. 81 (cumulative incidence 22%) BA.1, BA.1.1 and BA.2 infections were detected. High serum antibody titers are shown to be protective against infection (*p* < 0.01), linked to reduced viral load (*p* < 0.01) and time to viral clearance (*p* < 0.05). Pre-omicron SARS-CoV-2 infection is independently associated to increased protection against omicron, largely mediated by mucosal spike specific IgA responses (nested models lr test *p* = 0.02 and 0.008). Only 10% of infected participants remain asymptomatic through the course of their infection. We demonstrate that high levels of vaccine-induced spike-specific WT antibodies are linked to increased protection against infection and to reduced viral load if infected, and suggest that the additional protection offered by pre-omicron SARS-CoV-2 infection largely is mediated by mucosal spike-specific IgA.

## Abstract (web summary)

Correlation between vaccine induced serological response and protection against SARS-CoV-2 omicron infection is not well understood. Authors investigate breakthrough infections in triple-vaccinated healthcare workers, to characterise correlates of protection and viral characteristics.

## Introduction

The SARS-CoV-2 B.1.1.529 (omicron) variant has caused a considerable surge in COVID-19 cases, including in populations with high vaccine uptake^1^. While the now widely administered booster mRNA vaccine (third dose) have been shown to be effective against severe COVID-19^1,2^ caused by omicron, protection against infection appears limited and not sufficient to prevent viral transmission^3,4^. Vaccine induced serological responses correlate well to the risk of infection with the ancestral virus and pre-omicron SARS-CoV-2 variants of concern^5–8^, but less is known regarding the correlation between serological response and protection against omicron infection.

The omicron surge was initially caused by sublineages including BA.1, BA.1.1, and BA.2^9^. BA.2 carried a transmission advantage, and replaced BA.1 as the dominating sublineage in several countries^10^. Mutations in the spike protein distinguish the omicron sublineages from each other, but in vitro neutralization data suggest similar vaccine induced neutralizing capacity against BA.1 and BA.2^11^.

We investigated breakthrough infections in triple-vaccinated healthcare workers (HCW) with and without prior non-omicron SARS-CoV-2 infection during four weeks in January-February 2022, the first period of omicron transmission in Sweden. During the study period BA.1, BA.1.1 and BA.2 circulated in Stockholm, Sweden, allowing for comparison of breakthrough infections with the three sublineages^9^. The association between serum antibody levels, protection against infection and viral RNA trajectories were analyzed. In this work, we show a high cumulative incidence in BA.1, BA.1.1 and BA.2 breakthrough infections, with viral RNA trajectories suggestive of infectivity, five weeks after the vaccine booster dose. Increasing post booster serum antibody titers entailed a protective role against infection and had a reducing effect on viral load, independent of mucosal spike-specific IgA titers.

## Results

### High rates of Omicron breakthrough infection in triple-vaccinated HCW

**Fig. 1:**
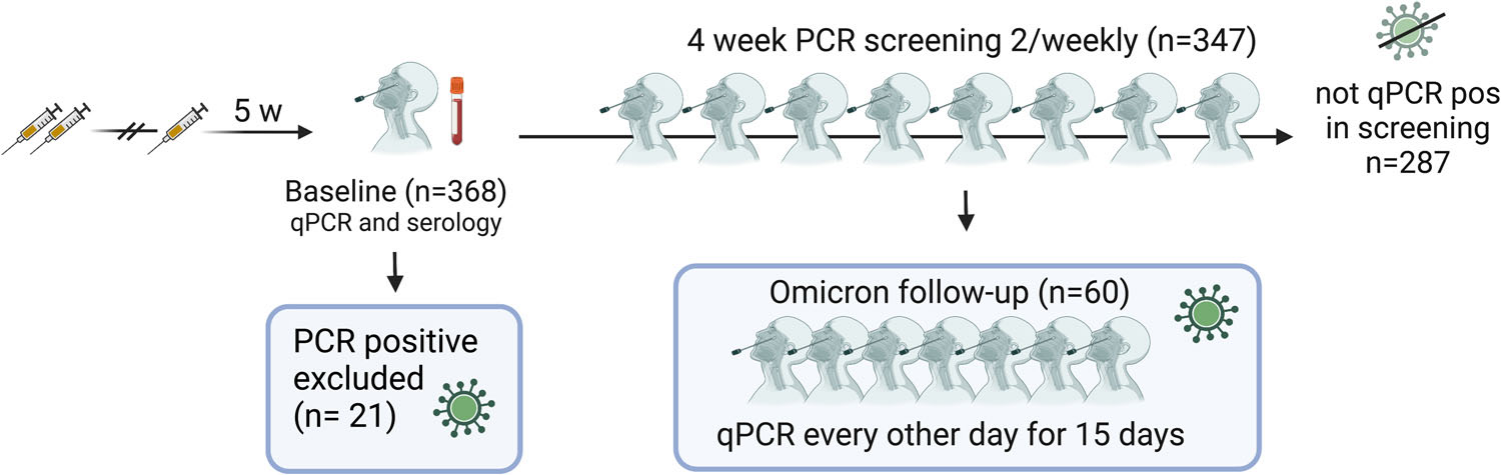
Overview of study cohort. Nasal/oropharyngeal/saliva swabs for PCR, mucosal antibody analysis^12^ and serum were collected at enrolment (baseline). Participants who tested qPCR positive at enrolment (*n* = 21) were excluded from all analyses except for estimation of cumulative incidence. Self-administered nasal/oropharyngeal/saliva swabs for qPCR were collected twice weekly for 4 weeks or until positive qPCR test from participants who tested qPCR negative at enrolment (*n* = 347). Participants who subsequently tested qPCR positive during the screening program (*n* = 60) were enrolled in an extended program comprising self-administered nasal/oropharyngeal/saliva swabs for qPCR every other day for 15 days.

To assess the risk of breakthrough infections with omicron following booster vaccination, 368 triple-vaccinated healthcare workers, naïve to SARS-CoV-2 omicron, were enrolled in a qPCR-screening study 5 weeks after their booster vaccine dose (3^rd^ dose), Fig. 1. Self-administered naso-oropharyngeal/saliva tests were performed twice weekly for four weeks (median adherence 2 samples per week, IQR 1.75-2). The total time-at-risk was 1324 person-weeks, total number of screening samples 2068 and complete case analysis applied.

**Fig. 2:**
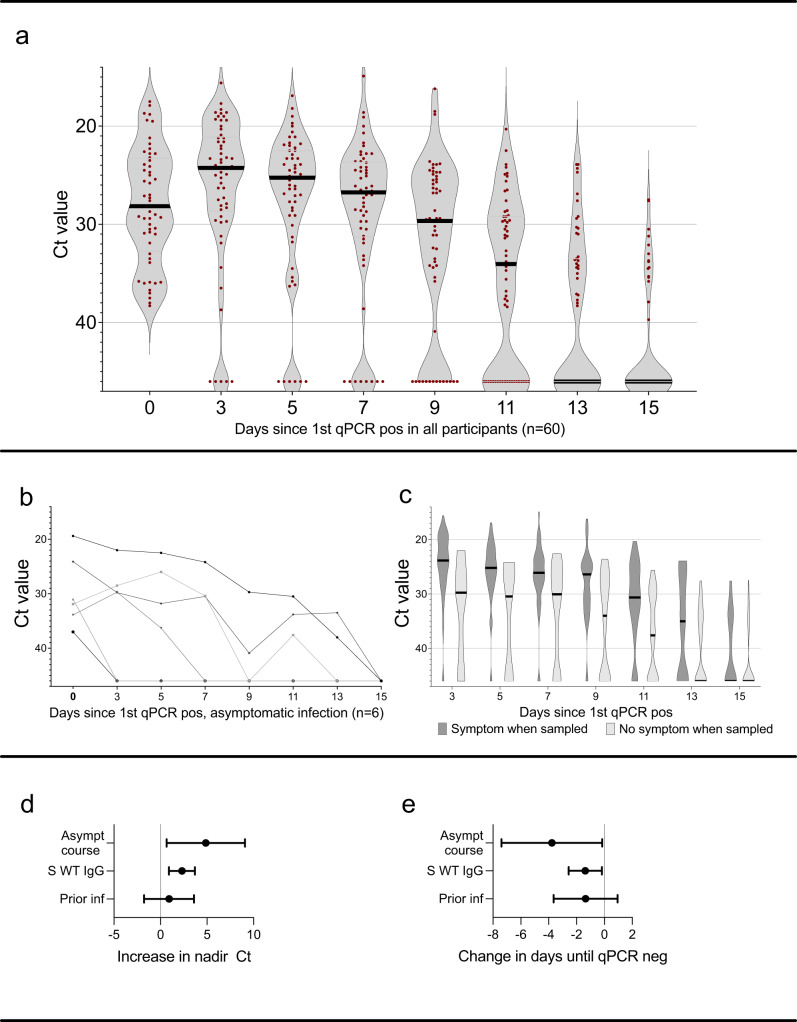
SARS-CoV-2 omicron viral load over the first 15 days of infection, symptom relation to Ct values and effects on nadir Ct and duration of qPCR positivity. **a** Ct value during the first 15 days of breakthrough infection in all qPCR positive participants (*n* = 60), and (**b**) in participants with an asymptomatic course of infection (*n* = 6). **c** Ct values in participants who were symptomatic (dark grey) or asymptomatic (light grey) at time of sampling. **d** Increase in nadir Ct by asymptomatic course of infection, per two-fold increase in WT spike-specific IgG levels and prior SARS-CoV-2 infection and (**e**) change in days until viral clearance (qPCR neg test) by asymptomatic course of infection, per two-fold increase in WT spike-specific IgG levels and prior SARS-CoV-2 infection. Estimates in **d** and **e** are derived from linear regression models and error bars depict 95% confidence interval. Ct Cyclic threshold, qPCR qualitative polymerase chain reaction, pos positive, asympt asymptomatic, S WT serum spike-specific wild type, inf infection. Source data are provided as a Source Data file.

A total of 81 omicron breakthrough infections were detected during the four-week screening period (22% of all included participants (*n* = 368)), among which 21 were detected at study inclusion and excluded from further analysis except for estimation of cumulative incidence. Frequency of COVID-19 patient contact, non-COVID-19 patient contact or non-patient related work was similar among participants with and without omicron breakthrough infection (Table S1). Sixty participants who were negative at first sample and subsequently tested positive during the four-week screening period were enrolled in a 14-day follow up with self-administered qPCR-samples every second day. Adherence to follow-up samplings was high with a median of 7 (IQR 7-7) self-administered follow-up samples. Viral RNA reached peak levels at day three after the initial positive test, and the majority of participants remained positive with Ct < 30 nine days after initial positive test (Fig. 2a). Five participants, all with Ct values > 30 in the initial positive sample, were qPCR negative in all follow-up samples. Six participants (10%) remained asymptomatic throughout the whole course of their infection, but, notably, three of these had Ct values < 30 for up to seven days (Fig. 2b).

More than one third, 23 of 60 (38%) participants, remained asymptomatic > 48 h after first qPCR-positive sample, with a median pre-symptomatic Ct value of 28.9 (range 19.4–38.0). Presence of symptoms at time of sampling were correlated to a higher viral load as compared to samples from asymptomatic participants (*p* < 0.0001, Mann-Whitney U-test) (Fig. 2c). Participants with an asymptomatic course of infection (*n* = 6) had a significantly higher nadir Ct value with an increase of 4.25 (95% CI 0.66–7.85, linear regression), (Fig. 2d), and shorter time to viral clearance (−3.36 days, 95% CI −6.56 to −0.15, linear regression), (Fig. 2e). Among symptomatic participants, “common cold” symptoms dominated (Fig. S1A).

Isolation of infectious viruses was successful from nine individuals (three infected with BA.1 and six with BA.1.1). Interestingly, isolation of viable viruses was possible from one sample with Ct 27 and from one sample collected at day 9, indicating that samples with relatively high Ct levels and samples from late in the course of infection may contain viable virus.

### High antibody titers are associated with protection and reduced viral load

#### Serum antibodies

**Fig. 3:**
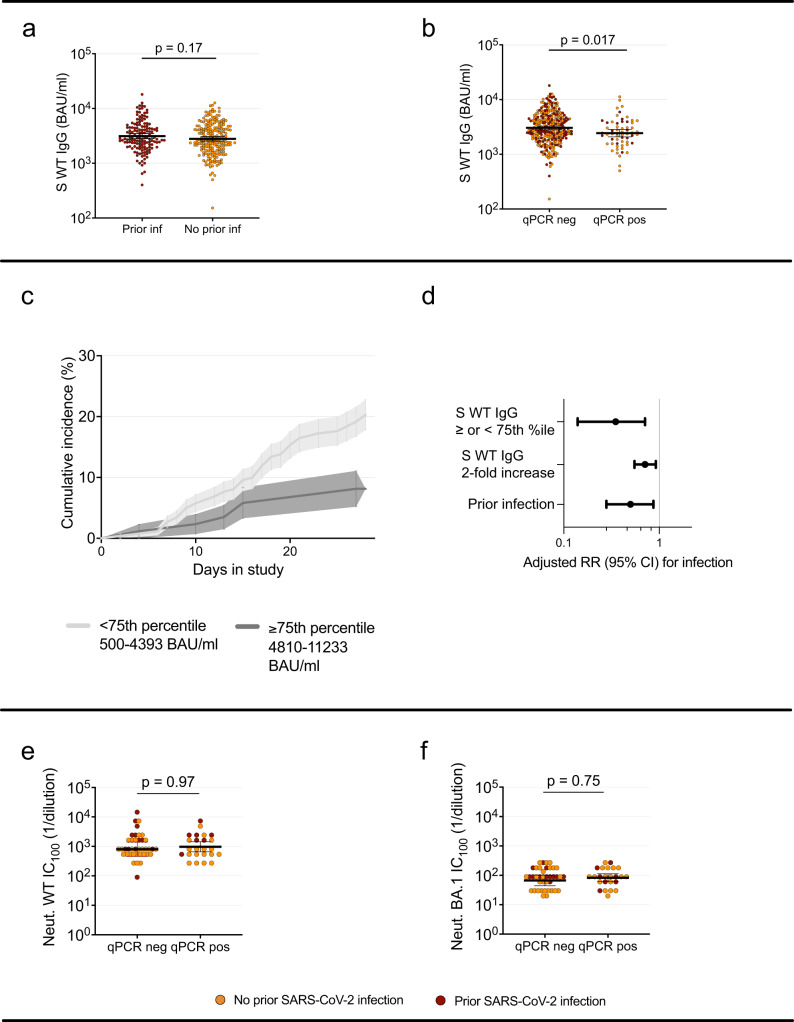
Effect of antibody levels and prior infection status on risk of infection, and pre-infection antibody titers in participants who subsequently tested qPCR positive or not. **a** WT spike-specific serum-IgG at baseline in participants with (*n* = 144) and without (*n* = 203) prior SARS-CoV-2 infection. **b** WT spike-specific serum IgG at baseline in participants that remained qPCR negative (*n* = 287) and participants that tested qPCR positive (*n* = 60) during the screening period. **c** Cumulative incidence over the study period in participants with spike-specific serum IgG levels above/at (*n* = 87) or below (*n* = 260) 75^th^ percentile. Shaded areas depict Standard Error. **d** Adjusted relative risk of infection among participants above (*n* = 87) vs below (*n* = 260) 75^th^ percentile, per two-fold increase in WT spike-specific serum-IgG level and among those with and without prior infection. Estimates derived from a Poisson regression model and error bars depict 95% confidence interval. **e** Microneutralization titers against SARS-CoV-2 WT and (**f**) omicron BA.1 in participants that remained qPCR negative (*n* = 40) and participants that tested qPCR positive (*n* = 24) during the screening period. In (**a**), (**b**), (**e**) and (**f**), Mann-Whitney U test with a two-tailed *p*-value was performed without adjustment for multiple comparisons. Lines depict geometric mean titer and bars depict 95% confidence interval. S spike, WT Wild type, RR relative risk, CI confidence interval, Ct cyclic threshold, qPCR qualitative polymerase chain reaction, pos positive, neg negative, BAU binding antibody units, Neut microneutralizing titer, ns; *p* > 0.05. Source data are provided as a Source Data file.

Baseline WT spike-specific serum IgG (serum-IgG) levels did not differ significantly between participants with (*n* = 144) and without (*n* = 203) prior SARS-CoV-2 infection (*p* = 0.17, Mann-Whitney) (Fig. 3a). Serum-IgG geometric mean titer (GMT) at inclusion were slightly lower among participants that subsequently tested positive (*n* = 60) and those that remained negative (*n* = 287) throughout the screening period, (GMT 3035 BAU/ml in the non-infected group vs 2432 BAU/ml among those subsequently infected, p = 0.017, Mann-Whitney U-test) (Fig. 3b). High levels of antibodies were associated to a reduced probability of testing qPCR positive during the study (*p* = 0.01, log-rank test) (Fig. 3c). To assess the potential protective effect of serum-IgG against omicron infection, we compared the risk of infection among participants with serum-IgG-levels above (*n* = 87) or below (*n* = 260) the 75^th^ percentile in a Poisson regression model. Adjusted relative risk of infection for participants above vs below 75^th^ percentile of serum-IgG was 0.35 (95% CI 0.14–0.71, Poisson regression) (Fig. 3d). Notably, increase in antibody level was linearly associated to increase in protection, with a RR of 0.71 (95% CI 0.55–0.92, Poisson regression) per two-fold increase in serum-IgG, (adjusted for age, sex and prior infection) (Fig. 3d). Adjusted relative risk of symptomatic infection were similar, 0.72 (95% CI 0.55–0.95, Poisson regression) per every two-fold increase in serum-IgG titer. Unadjusted estimates are provided in table S2.

High serum-IgG levels were linked to a reduced peak viral load and time to viral clearance, with an increase in Ct nadir by 2.37 (95% CI 0.99 to 3.76, linear regression) and a reduction of time to qPCR negativity by −1.37 days (95% CI −0.18 to −2.56, linear regression) per two-fold increase in serum-IgG, (Fig. 2d, e).

Participants with prior SARS-CoV-2 infection had a reduced risk of testing qPCR positive (adjusted RR 0.52, 95% CI 0.29 to 0.90, Poisson regression) (Fig. 3d). Prior SARS-CoV-2 infection was furthermore associated with a trend towards shorter time to viral clearance (−1.35 days (95% CI 0.95 to −3.65, linear regression)) (Fig. 2e) and a shorter duration of symptoms (Fig. S1B) but we found no significant effect on nadir Ct (increase by 0.92, 95% CI −1.77 to 3.60, linear regression) (Fig. 2d).

In the subset of samples (*n* = 64) where live virus microneutralization was performed, participants with prior infection (*n* = 19) had significantly higher neutralization titers against WT compared to those without prior infection (*n* = 45)(*p* < 0.01, Mann-Whitney U-test). There was however no significant difference in neutralizing titers against BA.1 between the groups (p 0.16, Mann-Whitney U-test) (Fig. S1C, D). We could not detect any difference in neutralizing titers against WT or BA.1 (Fig. 3e, f) between participants with (*n* = 24) and without (*n* = 40) subsequent breakthrough infection (*p* = 0.95 and 0.75, respectively, Mann-Whitney U-test). The neutralizing capacity was stronger against WT than against omicron in both groups (Figure S1E). Correlation between WT spike-specific IgG and live virus microneutralization of ancestral and omicron SARS-CoV-2 were equally high, both spearman r 0.64 (*p* < 0.0001) (Figure S1F, G). Although omicron neutralization titers were overall lower, live WT SARS-CoV-2 virus neutralization titers correlated to BA.1 sublineage neutralization titers (spearman r 0.52, *p* < 0.0001) (Figure S1H).

#### Mucosal antibodies’ role in protection against infection

We recently demonstrated an association between mucosal spike-specific IgA (mucosal IgA) and protection against omicron breakthrough infection in the same cohort^12,13^. To assess whether the here-in reported protective effect by high serum-IgG may be mediated through an IgG spill-over from serum to mucosa, or associated to the production of secretory IgA antibodies in the mucosa, the Poisson regression model was complemented with mucosal antibody levels. Interestingly, addition of mucosal IgG or mucosal IgA did not change the risk estimates associated to serum-IgG. However, in nested models, mucosal IgG had no effect while subtraction of mucosal IgA reduced the model fit (AIC 425 vs 420, likelihood ratio test *p* = 0.0008). The protective effect from prior infection dropped from 0.52 (95% CI 0.29–0.90) to 0.71 (95% CI 0.38–1.26), after introducing mucosal IgA, suggesting mucosal IgA to be a mediator of the prior infection effect. Taken together, these findings may suggest that while high serum-IgG titers protect against infection regardless of mucosal immune responses, the additive protective effect associated to prior infection is largely mediated through mucosal IgA and not by serum-IgG.

### Comparisons between omicron BA.1, BA.1.1, and BA.2 sub-lineages breakthrough infections

**Fig. 4:**
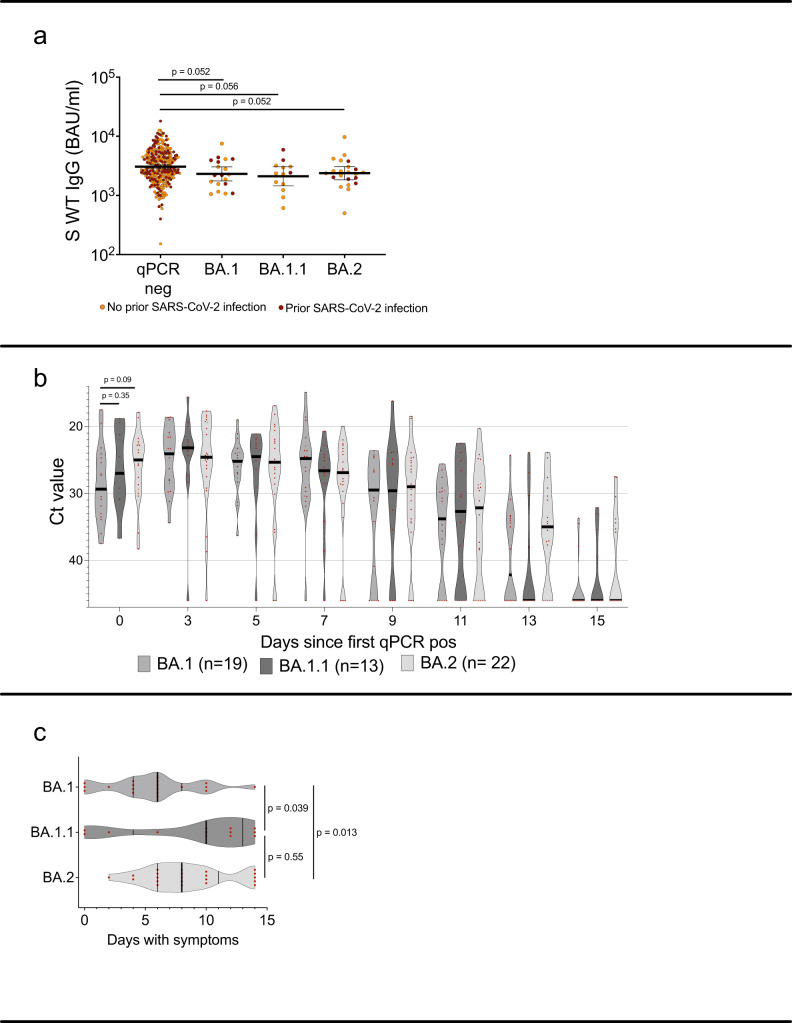
Post booster WT spike-specific IgG levels with comparisons between subsequent omicron sublineage breakthrough infections. **a** Post booster WT spike-specific IgG titers in participants that remained qPCR negative (*n* = 287) and in participants who tested positive with BA.1 (*n* = 19), BA1.1 (*n* = 13) or BA.2 (*n* = 22) infection during the screening period. **b** Ct values in qPCR positive participants the first 15 days of breakthrough infection with BA.1, BA.1.1 and BA.2. **c** Number of symptomatic days for participants who tested positive with BA.1, BA1.1 or BA.2 infection during the screening period. In (**a**), lines depict geometric mean titer and bars depict 95% confidence interval. In (**b**) and (**c**) lines depict median with IQR. Mann-Whitney U test with a two-tailed *p*-value was performed without adjustment for multiple comparisons. Pos Positive, Neg negative, S spike, WT wild-type, Ct cyclic threshold, BAU binding antibody units, ns; *p* > 0.05. Source data are provided as a Source Data file.

Whole genome sequencing was successful in 70/71 cases with at least one sample with Ct < 35, identifying 25 BA.1 (of which 19 were included in follow-up and analysis), 21 BA.1.1 (13 in follow-up and analysis), and 24 BA.2 (22 in follow-up and analysis) infections. There was a non-significant trend towards lower pre-infection WT spike-specific IgG levels among participants who subsequently became infected, and those that remained qPCR negative, in all omicron sublineages (Fig. 4a).

Median Ct value of first positive sample was 29.4 in BA.1 vs. 24.5 in BA.2 infections (Fig. 4b), corresponding to an approximate 100-fold higher level of viral RNA in BA.2 infected individuals early in the course of infection. These differences were however not significant (*p* = 0.09 Mann-Whitney U-test). We could not identify any strain-dependent variations in nadir Ct or time to viral clearance (linear regression models with nadir Ct or time to viral clearance as dependent variable, *p* > 0.2 and *p* > 0.6, respectively). Duration of symptoms was prolonged in BA.2- compared to BA.1- infected individuals, with median duration of symptoms 8 vs 6 days (*p* < 0.05 Mann-Whitney U-test), respectively (Fig. 4c). There were no asymptomatic cases among BA.2 infections (*n* = 22).

## Discussion

We report a high rate of breakthrough infections (22% over a period of 4 weeks) in a cohort of healthcare workers recently receiving an mRNA booster immunization and with a high rate of prior infection, supporting previous in vitro^14–16^ and epidemiological^2,10^ reports of omicron immune evasion. High post-booster serum-IgG titres displayed a protective effect and ameliorated viral load in those infected with omicron, contributing to the demonstrated short-term mRNA booster effectiveness against subsequent omicron infection and onward transmission^3,10^.

Although identification of SARS-CoV-2 RNA through qPCR is not equivalent to the detection of infectious virus, low Ct values have repeatedly been shown to correspond to viable virus in cell cultures for both omicron^17,18^ and other SARS-CoV-2 variants^19,20^. Consequently, Ct values are often used as a proxy of viral load. Fall et al. reported presence of infectious omicron virus in samples obtained up to eight days after symptom onset. The presence of infectious omicron virus was similar in non-vaccinated, vaccinated and boosted individuals^18^, implying that vaccine has little effect on viral load once infected. This is a highly relevant difference from findings related to previous SARS-CoV-2 variants^21,22^. In line with this, we demonstrate high viral RNA levels up to nine days after first qPCR positive sample, including after symptom resolution, in the majority of omicron breakthrough infections occurring shortly after a vaccine booster dose. Together, this suggests that recently vaccinated omicron-infected individuals may transmit the virus for a longer time period than the five days quarantine from symptom onset recommended by current guidelines^23,24^. This is of particular importance in vulnerable environments such as healthcare settings. Our data are furthermore in line with a report demonstrating a peak in omicron viral load two to five days after symptom onset^17^, where virus isolation was positive in 19% of vaccinated but not boosted infected individuals nine days after first positive qPCR test.

Asymptomatic and pre-symptomatic transmission play important roles in transmission dynamics. Accurate estimations of the number of asymptomatic infections are key in mathematic modelling and assumptions of population immunity. No more than 10% of cases in our study had an entirely asymptomatic course of infection, contradicting an early report suggesting a high rate of asymptomatic omicron infection^25^. Importantly, although Ct values were generally higher among asymptomatic and pre-symptomatic participants, several asymptomatic cases displayed low Ct values, emphasizing the role of asymptomatic transmission also in populations with a high vaccine uptake.

The previously reported increase in serum-IgG levels following primary vaccination in participants with prior infection^26,27^, were diminished after the booster dose. Interestingly, however, despite similar serum-IgG levels, infection prior to vaccination conferred additional protection against omicron breakthrough infection. Live virus BA.1 neutralization did not differ significantly between participants with and without prior infection and it is likely that other mechanisms, such as mucosal immune responses contribute. Our analysis shows that the additive protection conferred by prior infection largely may be mediated through mucosal spike-specific IgA^12^. Furthermore, the protective effect of serum-IgG is not associated to mucosal IgA levels. This indicates independent mechanisms of protection in these two compartments, and questions the use of systemic antibody responses alone as markers of protection against omicron breakthrough infection. Stronger protection against pre-omicron SARS-CoV-2 infection with increasing antibody levels has been brought forward by several reports^5,5,8,22,28^. Feng et al. found 80% protection against SARS-CoV-2 alpha infection at an spike-specific IgG level of 247 BAU/ml^28^ in the ChAdOx1 study cohort, and review of data from other vaccine trials suggest correlates of similar levels^8^. Although post booster serum-IgG levels were significantly associated with a lower risk of breakthrough infection in the regression model, the difference in geometric mean titer (GMT) of serum-IgG between the group that became infected and the group that remained negative was small and not significant in live virus neutralization titers (possibly due to limited sample size). It is noteworthy that serum-IgG GMTs of both groups were 10-fold higher than the level conferring an 80% protection against alpha^28^ (only one participant in our study cohort had post booster spike-specific IgG titer below 247 BAU/ml). The high cumulative incidence despite these comparatively high antibody levels in this group illustrates the immune evasive potential of omicron. The well documented rapid waning of antibody levels following both primary vaccination and booster doses^29–31^ likely renders it difficult to maintain this high antibody levels with current vaccine strategies.

This study is limited by the observational design, and that the cohort is comprised of predominantly young and healthy individuals with a female dominance. Study power was not large enough to detect differences between subgroups of infected participants, such as substrains of omicron. A potential confounder is differences in exposure risk, which is difficult to overcome in an observational study but may affect prior infection, vaccination history, and risk of infection. The study is however strengthened by the comprehensive screening program with high adherence to testing, thereby limiting the risk of missing transient and asymptomatic cases. Vaccine status and prior PCR-confirmed infection were obtained from high-quality national registries, and prior infection was also determined through regular serology in the cohort since the start of the COVID-19 outbreak.

In conclusion, identifying potential immune correlates of protection from infection and understanding the kinetics of SARS-CoV-2 omicron shedding in vaccinated individuals is crucial to guide infection control measures and vaccination policy. We show a high incidence of omicron infection in a recently triple vaccinated health care worker cohort. These breakthrough infections were associated with high viral load, which likely contributes to the global surge in cases. The very high cumulative incidence despite a recent booster vaccine dose questions the relation between the detection of vaccine induced serum antibody levels and omicron risk prediction.

## Methods

This study was approved by the Swedish Ethical Review Authority (dnr 2020-01653) and conducted in accordance with the declaration of Helsinki. Written informed consent was obtained from all study participants.

### Study cohorts

The COMMUNITY study comprises 2149 HCW at Danderyd Hospital, Stockholm, Sweden, enrolled between April and May 2020. Study participants are followed every four months since inclusion^26,32–34^ with blood samples and collection of relevant information (such as chronic medication/immune suppression, work related SARS-CoV-2 exposure, etc.) through a smart phone-based application. SARS-CoV-2 infection prior to vaccination was confirmed by seroconversion at any of the follow-up visits and/or by PCR. All HCW were offered vaccination with either BNT162b2 (BNT) or ChAdOx1 nCoV-19 (ChAd), depending on availability, starting in January 2021. Data regarding which vaccine and date of vaccination is obtained through the Swedish vaccination register (VAL Vaccinera) and data regarding PCR-confirmed SARS-CoV-2 infection is obtained through the national communicable disease surveillance register SmiNet (Swedish Public Health Agency).

To investigate the risk of omicron breakthrough infections in triple vaccinated participants, and viral characteristics if infected, we invited 368 recently boosted (3^rd^ dose), SARS-CoV-2 omicron naïve, participants to a twice-weekly qPCR screening with self-administered naso-oropharyngeal/saliva swabs^35^ for four weeks. Inclusion in this screening study was conducted in conjunction to the sixth follow-up in January 2022. All participants who had completed primary vaccination and received a BNT or a MOD booster were invited (*n* = 802), of which the first 368 participants who came to the sixth follow-up and conceded to the screening study were included. The study cohort is presented in Fig. 1 and demographics in Table 1. Participants with qPCR confirmed SARS-CoV-2 infection between their third vaccine dose and appointment for study inclusion were not included in the study. SARS-CoV-2 omicron became dominant in Stockholm two weeks before study inclusion. Serum and mucosal antibody levels were determined at study inclusion. Participants testing positive at the inclusion qPCR test (*n* = 21) were excluded from further analysis but included in estimation of cumulative incidence. Positive qPCR tests after a negative inclusion test were followed by an extended set of self-administered swabs for qPCR every second day for 15 days post first positive sample. All study participants who engaged in the extended follow-up responded to a questionnaire including a pre-defined set of symptoms (fever, sore throat, cough, headache, anosmia and rhinorrea). After completing 15 days of follow-up sampling, participants continued in the twice-weekly screening until the end of the study period. qPCR, whole genome sequencing and virus isolation was performed as previously described^36^.

**Table 1:** Demographics, frequency of prior SARS-CoV-2 infection and vaccine regimen of study cohort

	Omicron pos (*N* = 60)	Not Omicron pos (*N* = 287)	All (*N* = 347)
**Age**			
Median (IQR)	51 (40–58)	54 (46–60)	53 (45–60)
**Sex**			
Female	55 (92%)	255 (89%)	310 (89%)
Male	5 (8%)	32 (11%)	37 (11%)
**Prior infection**			
No	43 (72%)	160 (56%)	203 (58%)
Yes	17 (28%)	127 (44%)	144 (42%)
**Primary Vaccine Regimen**			
BNT x 2	36 (60%)	182 (64%)	218 (63%)
ChAd + BNT	16 (27%)	58 (20%)	74 (21%)
ChAd x 2	8 (13%)	47 (16%)	55 (16%)
**Booster vaccine**			
MOD	45 (75%)	218 (76%)	263 (76%)
BNT	15 (25%)	69 (24%)	84 (24%)
**Days 3rd vaccine to inclusion**			
Median (IQR)	34 (31-36)	34 (32-37)	34 (32-37)

*IQR* Interquartile range, *pos* Positive, *MOD* mRNA-1273 vaccine, *BNT* BNT162b2 mRNA vaccine.

### Serological and mucosal antibody investigation

SARS-CoV-2 WT spike-specific IgG were measured in post booster (3rd dose) samples drawn at start of the screening study (V-PLEX SARS-CoV-2 Panels 23 and 25, Meso Scale Diagnostics, Maryland, USA). WT spike-specific IgG titers are expressed in the WHO-standard binding antibody units (BAU)/ml.

Mucosal spike-specific SARS-CoV-2 IgA and IgG were determined as previously described using V-PLEX SARS-CoV-2 Panels 24 and 25^12^.

### Live-virus microneutralization

In order to establish correlations to binding antibody assays, a sub-set of participants were tested for neutralizing capacity against SARS-CoV-2 WT and omicron BA.1 using a micro-neutralization assay as previously described^37^. Samples were stratified by primary vaccine regimen and 64 (maximum capacity) samples randomly selected, (45 SARS-CoV-2 naïve and 19 recovered). Briefly, heat-inactivated serum was 3-fold serially diluted, mixed with virus, incubated for 1 h and finally added, in duplicates, to confluent Vero E6 cells (bought from ATCC Cat# CRL-1586, RRID: CVCL_0574) in 96-well plates. Original SARS-CoV-2 WT and omicron BA.1 (both isolated from Swedish patients) were used. After 5 days incubation, the wells were inspected for signs of cytopathic effect (CPE) by optical microscopy. Serum neutralizing activity was measured by 100% CPE inhibition (IC_100_). Each well was scored as either neutralizing (if no signs of CPE was observed) or non-neutralizing (if any CPE was observed). The arithmetic mean neutralization titer of the reciprocals of the highest neutralizing dilutions from the two duplicates for each sample was then calculated.

### Virus isolation

All respiratory samples were added to confluent Vero E6 cells and incubated for 10 days with regular exchange of medium. Viral infectivity was assessed manually by microscopy (signs of CPE) and was confirmed by qPCR (lower Ct value in supernatants over time, showing active viral replication).

### Statistics

Risk of breakthrough infection over the four weeks screening period was evaluated using a Poisson regression model with a log offset for observed time. Time was defined as weeks until infection or study end. We first investigated the difference in risk of infection among participants with serum spike-specific IgG levels ≥ 75^th^ percentile and < 75^th^ percentile, and secondly the change in risk of infection for every two-fold increase in serum IgG levels, while adjusting for prior infection status, age and sex. Analyses were performed with and without the addition of serum-IgA and mucosal antibody IgA/IgG-levels above (including 75^th^ percentile) or below 75^th^ percentile. There was no support of non-linearity or the need of applied interaction between prior infection status and serum antibody levels (*p* > 0.9). Nested models were compared with likelihood ratio test and relevant variables (prior infection, mucosal IgA) were evaluated with AIC to compare fits.

Linear regression models were generated for nadir Ct levels and for number of days until negative qPCR, both adjusted for age, sex, prior infection status, log2-transformed WT spike-specific serum IgG levels and occurrence of asymptomatic course of infection. Since whole genome sequencing was performed only if Ct < 30, Ct variations between different strains were investigated in a separate regression model with same adjustments. For statistical comparisons, negative qPCR-samples were given a Ct value of 46. Mann-Whitney U test was performed for comparisons of antibody titers between groups, correlations were estimated by non-parametric Spearman correlation test. Statistical analyses were performed using GraphPad Prism version 9.2.0 (GraphPad Software, San Diego, California, USA) or statistical program R (2022.07.1 + 554 “Spotted Wakerobin” Release), using packages nlme, Greg, contrast, tidyverse (RStudio Team 2019, Boston, USA).

### Reporting summary

Further information on research design is available in the Nature Portfolio Reporting Summary linked to this article.

## Supplementary information

Supplementary Information

Reporting Summary

## Data Availability

Source data are provided with this paper (DOI:10.5281/zenodo.7585587). The anonymized datasets generated during and/or analyzed during the current study are available from the corresponding author upon reasonable request. Requests for raw and analyzed data will be promptly reviewed by the corresponding author (C.T.) to determine if they are subject to intellectual property or confidentiality obligations. Any data and materials that can be shared will be released via a material transfer agreement (requested to C.T.). Personal data underlying this article cannot be shared publicly as they are sensitive. Enquiries regarding data availability should be directed to charlotte.thalin@ki.se. Source data are provided with this paper.

## Source data

Source Data

## Code availability

All the codes used for processing and analyzing the data in this study have been deposited in an available GitHub repository (DOI: 10.5281/zenodo.7404567)
