# Supplementary material for: Correlates of protection and viral load trajectories in omicron breakthrough infections in triple vaccinated healthcare workers
Source: Nat Commun. 2023 Mar 22;14:1577. doi: 10.1038/s41467-023-36984-1 (PMC10031702; doi:10.1038/s41467-023-36984-1)
Supplement: Supplementary file 1 — Supplementary Information [file 41467_2023_36984_MOESM1_ESM.docx]

**Supplemental figures and tables**

**Correlates of protection and viral load trajectories in omicron breakthrough infections in triple vaccinated healthcare workers**

Ulrika Marking, Sebastian Havervall, Nina Greilert Norin, Oscar Bladh, Wanda Christ, Max Gordon, Henry Ng, Kim Blom, Mia Phillipson, Sara Mangsbo, Jessica J. Alm, Anna Smed-Sörensen, Peter Nilsson, Sophia Hober, Mikael Åberg, Jonas Klingström, Charlotte Thålin

| Type of work | Tested qPCR pos at inclusion or in study  (N=81) | Did not test qPCR pos  (N=287) | Total  (N=368) |
| --- | --- | --- | --- |
| COVID-19 patient contact, n (%) | 35 (43%) | 138 (48%) | 173 |
| Non-COVID-19 patient contact, not designated COVID-19 wards, n (%) | 33 (41%) | 105 (37%) | 138 |
| Not patient related work, n (%) | 10 (12%) | 39 (13%) | 49 |
| Data missing n (%) | 3 (4%) | 6 (2%) | 9 |

**Table S1.** Frequency of patient related work, accounting for possible differences in SARS-CoV-2 exposure, between health care workers with and without a positive qPCR test.

|  | **Coef** | **95% CI** |
| --- | --- | --- |
| S-IgG (per 2-fold increase) | 0.76 | 0.59 to 0.97 |
| Prior infection | 0.53 | 0.29 to 0.90 |

**Table S2**. Crude estimates of risk of Omicron infection, derived from univariate poisson regression model. Coef; coefficient, CI; Confidence interval, S-IgG; Spike specific IgG (WT).


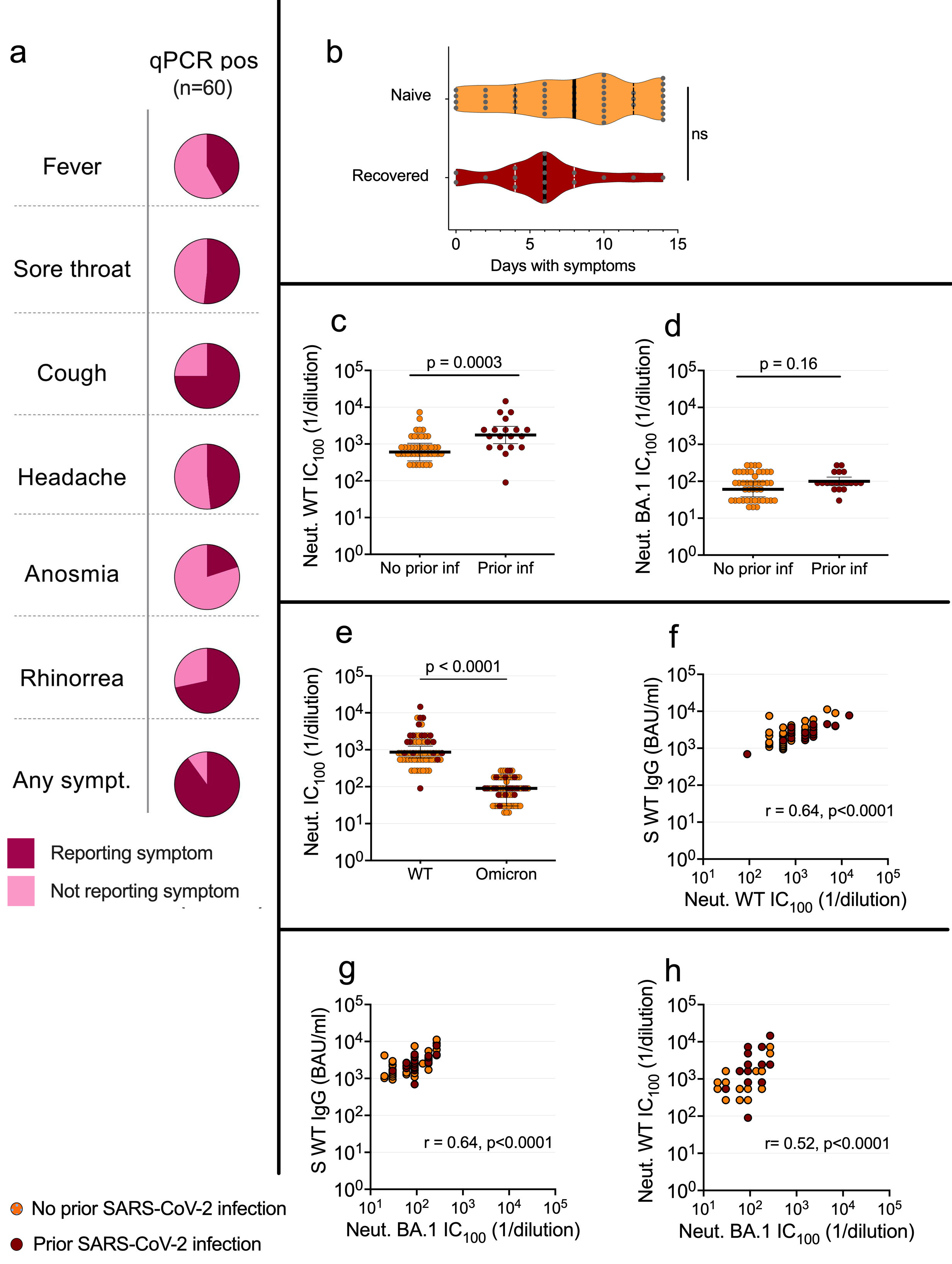


**Figure S1** **Self-reported symptomatology during omicron breakthrough infections and live-virus neutralization titers at start of PCR-screening study. (A)** Reported symptoms and prevalence of such in qPCR positive participants (n=60) with (purple) and without (pink) fever, sore throat, cough, headache, anosmia and rhinorrea. (**B**) Number of symptomatic days among infected participants with (dark red, n=17) and without (orange, n=43) prior infection. Baseline neutralizing titers against **(C)** WT and **(D)** omicron strains in participants with (n=19) and without (n=45) prior infection. **(E)** Comparison of neutralizing titers against WT and omicron strains. Correlation of WT spike-specific IgG titers to **(F)** WT and **(G)** omicron live-virus neutralizing titers, and **(H)** correlation of WT and omicron neutralizing titers. In (B-E), Mann-Whitney U test with a two-tailed p value was performed without adjustment for multiple comparisons. Lines depict geometric mean titer and bars depict 95% confidence interval. In (F-H), r-values are obtained from Spearmans correlation test. qPCR; qualitative polymerase chain reaction, pos; positive, sympt; symptom. Pos; positive, neg; negative, S; spike, WT; wild-type; BAU; binding antibody units, neut; neutralizing, inf; infection, ns; p > 0.05. Source data are provided as a Source Data file.
