# Supplementary material for: Correlates of protection and viral load trajectories in omicron breakthrough infections in triple vaccinated healthcare workers
Source: Nat Commun. 2023 Mar 22;14:1577. doi: 10.1038/s41467-023-36984-1 (PMC10031702; doi:10.1038/s41467-023-36984-1)
Supplement: Supplementary file 2 — Reporting Summary [file 41467_2023_36984_MOESM2_ESM.pdf]

nature portfolio

Corresponding author(s): Charlotte Thålin

Last updated by author(s): Feb 5, 2023

Reporting Summary

Nature Portfolio wishes to improve the reproducibility of the work that we publish. This form provides structure for consistency and transparency in reporting. For further information on Nature Portfolio policies, see our Editorial Policies and the Editorial Policy Checklist.

Statistics

For all statistical analyses, confirm that the following items are present in the figure legend, table legend, main text, or Methods section.

n/a	Confirmed	
☐	☒	The exact sample size (*n*) for each experimental group/condition, given as a discrete number and unit of measurement
☐	☒	A statement on whether measurements were taken from distinct samples or whether the same sample was measured repeatedly
☐	☒	The statistical test(s) used AND whether they are one- or two-sided<br>*Only common tests should be described solely by name; describe more complex techniques in the Methods section.*
☐	☒	A description of all covariates tested
☐	☒	A description of any assumptions or corrections, such as tests of normality and adjustment for multiple comparisons
☐	☒	A full description of the statistical parameters including central tendency (e.g. means) or other basic estimates (e.g. regression coefficient) AND variation (e.g. standard deviation) or associated estimates of uncertainty (e.g. confidence intervals)
☐	☒	For null hypothesis testing, the test statistic (e.g. *F*, *t*, *r*) with confidence intervals, effect sizes, degrees of freedom and *P* value noted<br>*Give P values as exact values whenever suitable.*
☒	☐	For Bayesian analysis, information on the choice of priors and Markov chain Monte Carlo settings
☒	☐	For hierarchical and complex designs, identification of the appropriate level for tests and full reporting of outcomes
☐	☒	Estimates of effect sizes (e.g. Cohen's *d*, Pearson's *r*), indicating how they were calculated

Our web collection on statistics for biologists contains articles on many of the points above.

Software and code

Policy information about availability of computer code

Data collection	No commercial, open source or custom code was used to collect the data in this study.
Data analysis	Statistical analyses were performed in RStudio, R version 4.2.1, (2022.07.1+554 "Spotted Wakerobin" Release (7872775ebddc40635780ca1ed238934c3345c5de, 2022-07-22) for macOS, using packages nlme, Greg, contrast, tidyverse (RStudio Team 2019, Boston, USA). Illustrations were performed in Graph Pad Prism. All the codes used for processing and analyzing the data in this study have been deposited in an available GitHub repository (DOI: 10.5281/zenodo.7404567).

For manuscripts utilizing custom algorithms or software that are central to the research but not yet described in published literature, software must be made available to editors and reviewers. We strongly encourage code deposition in a community repository (e.g. GitHub). See the Nature Portfolio guidelines for submitting code & software for further information.

Data

Policy information about availability of data

All manuscripts must include a data availability statement. This statement should provide the following information, where applicable:

- Accession codes, unique identifiers, or web links for publicly available datasets
- A description of any restrictions on data availability
- For clinical datasets or third party data, please ensure that the statement adheres to our policy

The anonymized datasets generated during and/or analyzed during the current study are available from the corresponding author upon reasonable request. Source data are provided with this paper (DOI:10.5281/zenodo.7585587).

Human research participants

Policy information about studies involving human research participants and Sex and Gender in Research.

Reporting on sex and gender	Female dominance of study cohort, sex adjusted for in regression analyses. No gender aspects applicable.
Population characteristics	Health care workes, reasonably representative of the working age population in Sweden, but with a female dominance. Median age 53 (IQR 45-60), prior SARS-CoV-2 infection 42%, primary vaccine regimen BNT162b2 (63%), ChAdOx1 (16%), heterologous BNT162b2/ChAdOx1 (21%).
Recruitment	Conveniance sampling at a scheduled COMMUNITY study follow-up. The first 368 (maximum laboratory capacity) participants agreeing to participate was included in this substudy. The offer to participate did thereby not reach all study participants in the main COMMUNTY cohort, and the sample in this sub cohort was not randomised. However, randomisation in this setting had been very unpractical, and we do not believe conveniance sampling in this manner confers bias. There is a possibility that participants with known high exposure to a larger extent chose to participate in this sub study but over all incidence estimates where not a main aim. For analysis of protective factors a high cumulative incidence is of large value.
Ethics oversight	The study was aproved by the Swedish Ethical Review Authority (dnr 2020-01653) and conducted in accordance with the declaration of Helsinki. Written informed consent was obtained from all study participants.

Note that full information on the approval of the study protocol must also be provided in the manuscript.

Field-specific reporting

Please select the one below that is the best fit for your research. If you are not sure, read the appropriate sections before making your selection.

☒ Life sciences ☐ Behavioural & social sciences ☐ Ecological, evolutionary & environmental sciences

For a reference copy of the document with all sections, see nature.com/documents/nr-reporting-summary-flat.pdf

Life sciences study design

All studies must disclose on these points even when the disclosure is negative.

Sample size	No sample size calculation was performed. The sample size was based on maximum capacity of PCR analyses. Due to the high viral transmission at the time of the screening study this number proved sufficient to achieve significance in protective estimates/viral characteristics.
Data exclusions	Participants with SARS-CoV-2 omicron infection prior to study start were not included and partcipants with positive qPCR at inclusion test were excluded from follow-up analyses within the study, since first day of infection could not be established.
Replication	Antibody data was generated using a commercial assay. Postive controls (low, median and high) was supplied by the manufacturer and analyzed in duplicates on each serology plate. qPCR assays were performed in a high security national reference laboratory with a validated method.
Randomization	The study is an observational study and there was no intervention. We therefore did not implement a randomization.
Blinding	Not applicable since there is no intervention in this study.

Reporting for specific materials, systems and methods

We require information from authors about some types of materials, experimental systems and methods used in many studies. Here, indicate whether each material, system or method listed is relevant to your study. If you are not sure if a list item applies to your research, read the appropriate section before selecting a response.

Materials & experimental systems

n/a	Involved in the study
☐	☒ Antibodies
☒	☐ Eukaryotic cell lines
☒	☐ Palaeontology and archaeology
☒	☐ Animals and other organisms
☐	☒ Clinical data
☒	☐ Dual use research of concern

Methods

n/a	Involved in the study
☒	☐ ChIP-seq
☒	☐ Flow cytometry
☒	☐ MRI-based neuroimaging

Antibodies

Antibodies used	Antibodies conjugated with SULFO-TAG were supplied by each kit and used as detection antibodies in the serology assays. LoT numbers for individual kits as well as the detection antibodies are available on the suppliers homepage https://www.mesoscale.com/en/products_and_services/assay_kits/covid-19
Validation	No primary antibodies was used in this study.

Clinical data

Policy information about clinical studies

All manuscripts should comply with the ICMJE guidelines for publication of clinical research and a completed CONSORT checklist must be included with all submissions.

Clinical trial registration	Not registered on ClinicalTrials.gov
Study protocol	Full study protocol can be accessed upon request to corresponding author.
Data collection	Consecutive study inclusion to main study took place between 15th of April to 8th of May 2020. Inclusion to this sub study took place jan 2022, first 368 omicron naïve (maximum capacity) participants consenting to participation were included.
Outcomes	Primary outcome was SARS-CoV 2 omicon infection, and relation to pre-infection antibody levels. Secondary outcome was viral characteristics such as duration of shedding, peak viral load.
